# Identification and Evolutionary Analysis of the Widely Distributed CAP Superfamily in Spider Venom

**DOI:** 10.3390/toxins16060240

**Published:** 2024-05-24

**Authors:** Hongcen Jiang, Yiru Wang, Guoqing Zhang, Anqiang Jia, Zhaoyuan Wei, Yi Wang

**Affiliations:** 1 Integrative Science Center of Germplasm Creation in Western China (CHONGQING) Science City, Biological Science Research Center, Southwest University, Chongqing 400715, China; jhc0607@email.swu.edu.cn (H.J.);; 2 Yazhouwan National Laboratory, Sanya 572024, China

**Keywords:** spider venom, CAP superfamily, Araneoidea toxin, venom evolution

## Abstract

Venom plays a crucial role in the defense and predation of venomous animals. Spiders (Araneae) are among the most successful predators and have a fascinating venom composition. Their venom mainly contains disulfide-rich peptides and large proteins. Here, we analyzed spider venom protein families, utilizing transcriptomic and genomic data, and highlighted their similarities and differences. We show that spiders have specific combinations of toxins for better predation and defense, typically comprising a core toxin expressed alongside several auxiliary toxins. Among them, the CAP superfamily is widely distributed and highly expressed in web-building Araneoidea spiders. Our analysis of evolutionary relationships revealed four subfamilies (subA-subD) of the CAP superfamily that differ in structure and potential functions. CAP proteins are composed of a conserved CAP domain and diverse C-terminal domains. CAP subC shares similar domains with the snake ion channel regulator svCRISP proteins, while CAP subD possesses a sequence similar to that of insect venom allergen 5 (Ag5). Furthermore, we show that gene duplication and selective expression lead to increased expression of CAP subD, making it a core member of the CAP superfamily. This study sheds light on the functional diversity of CAP subfamilies and their evolutionary history, which has important implications for fully understanding the composition of spider venom proteins and the core toxin components of web-building spiders.

## 1. Introduction

There are more than 50,000 species of spiders in the world that are distributed in 138 families [1]. Araneae is divided into Mesothelae and Opisthothelae. Mesothelae only includes one extant family, the Liphistiidae. Opisthothelae can be further divided into Mygalomorphae and Araneomorphae, with the latter accounting for about 90% of the natural spiders [2]. Araneomorphae can be classified into two main branches: Araneoidea and RTA (Retrolateral tibial apophysis) clade spiders. Spiders are natural predators of many agricultural pests and mainly feed on insects. Most spiders have efficient and complex venom to aid in their hunting, such as the Chinese bird spider (Mygalomorphae), Sydney funnel-web spider (Mygalomorphae), wandering spiders (RTA), brown spiders (RTA), and black widow spiders (Araneoidea).

Spider venom is composed of different components that can be classified into four main groups: inorganic salts, small-molecule compounds (<1 kDa), disulfide-rich peptides (DRPs), and large proteins (>10 kDa) [3]. Over 100 spider toxins have been identified and reported so far [4]. For instance, Lycosin-I, a short cationic peptide, has multifunctional properties, including antibacterial [5,6,7], antineoplastic [8], and anti-inflammatory [9] activities. The cysteine-rich theraphotoxin stimulates the pain-sensing neurons by enhancing sodium currents and decreasing potassium currents [10]. The large protein latrotoxin can regulate ion channels through calcium-dependent and -independent mechanisms of pore formation [11]. Additionally, phospholipase D exhibits insecticidal activity and can induce skin necrosis [12]. Toxicity varies among different spider species, and they seem to have their respective dominant toxins that help them adapt to different ecology.

The CAP superfamily is a large venom protein family, also known as the cysteine-rich secretory proteins (CRISPs), antigen 5 (Ag5), and pathogenesis-related 1 (PR-1) superfamily, which is named after the first initials of the three proteins it includes [13]. This family is found in many venomous animals, such as reptiles (snakes [14,15,16,17], lizards [18,19,20]) and arthropods (bees [21], ants [22], spiders [23,24,25,26], scorpions [27]), as well as bacteria [28], plants [29], fungi [30], and mammals [31].

The complete CAP protein consists of three domains. The first domain is the CAP domain (also known as the SCP domain/PR-1 domain), which comprises around 160 amino acids [13]. It contains four highly conserved feature motifs, namely, CAP3: HNxxR, CAP4: G[EQ]N[ILV], CAP1: [GDER][HR][FYWH][TVS][QA][LIVM][LIVMA]Wxx[STN], and CAP2: [LIVMFYH][LIVMFY]xC[NQRHS]Yx[PARH]x[GL]N[LIVMFYWDN]. The second domain is the hinge domain, which is about 20 amino acids long and contains two pairs of conserved disulfide bonds. The main purpose of this domain is to stabilize the spatial conformation between the CAP domain and the ion channel regulator (ICR) domain. The third domain is the ICR domain, which is made up of approximately 40 amino acids and has three pairs of conserved disulfide bonds. It has potential ion channel regulation capabilities, although the exact site of action is not yet clear. The hinge domain and ICR domain are rich in cysteine residues and therefore can be collectively referred to as the cysteine-rich domain (CRD) [28].

The CAP superfamily has diverse structures which allow it to perform a variety of functions. In yeast, the CAP protein is involved in lipid export, mating, and pathogen defense [32]. In plants, the CAP protein plays a crucial role in defending against pathogens by triggering immune responses and inducing cell death [29]. In mammals, the CAP protein is closely related to various aspects of fertility [31], such as sperm maturation and sperm egg fusion [33]. In venomous animals, the CAP protein has numerous functions, including ion channel inhibition [28,34,35,36], proinflammatory effects [37,38], antiangiogenesis [16,39], myotoxicity [40], antiprotozoal activity [14], and antimicrobial activity [41]. The CAP protein is also one of the venom allergens in Hymenoptera [22]. The CAP protein is found in spiders at the transcriptomic or proteomic level [23,24,25], and is especially abundant in the venom of the web-building spider *Argiope bruennichi* [26].

The CAP superfamily has been reported with numerous functions in a variety of venomous animals; however, its role and evolution in spiders remain unexplored. The recruitment process of this ancient, conserved venom protein family into spider venom and the function it serves are currently unknown.

Here, we present an analysis of spider venom from two different aspects. Firstly, we analyze the composition of spider venom protein families and identify their similarities and differences. We propose that there are conserved venom proteins and highly variable neurotoxins. They perform the basic functions of venom and help spiders adapt to their ecology. It is crucial to study specific spider toxins to treat spider bites and develop practical applications [42]. In the following sections, we analyze the multifunctional CAP superfamily. We find that spiders have a more diverse variety of CAP proteins than other venomous animals. Our sequence similarity analysis shows that the spider CAP superfamily has the potential to cause allergic reactions and regulate ion channels. Additionally, the CAP subD of the CAP superfamily is significantly expanded in Araneoidea, making the CAP superfamily a core toxin in these spiders. This helps us understand the toxins of web-building spiders that are often overlooked.

## 2. Results

### 2.1. The Similarities and Differences of Spider Venom Protein Families

Koua and Kuhn-Nentwig categorized spider toxins into 64 superfamilies, including 5 spider short linear cationic peptides (SCs), 19 venom proteins (VPs), and 40 spider neurotoxins (SNs) [43]. To comprehensively understand the distribution of these superfamilies in Araneae, we conducted a statistical analysis using publicly available transcriptome data [44]. A total of 74 spiders with the highest BUSCO completeness were selected from each family (Table S1). This dataset covers almost the entire spider tree of life, covering both primitive and ancient Mesothelae and Mygalomorphae, as well as the relatively new evolutionary status of Araneomorphae. The web-building Araneoidea spiders and the hunting-living RTA clade spiders are the two most abundant species in this dataset. The study of toxins has mainly focused on a few groups such as Theraphosidae, Hexathelidae, Scytodoidea, and Lycosoidea. Through the comprehensive identification and statistical analysis of venom protein families in Araneae (Table S2), we reported the toxin transcripts of spiders that have never been reported in public databases. And for those spiders that have only analyzed specific peptides in their venom, our findings provide a detailed complement to their toxin composition.

**Figure 1:**
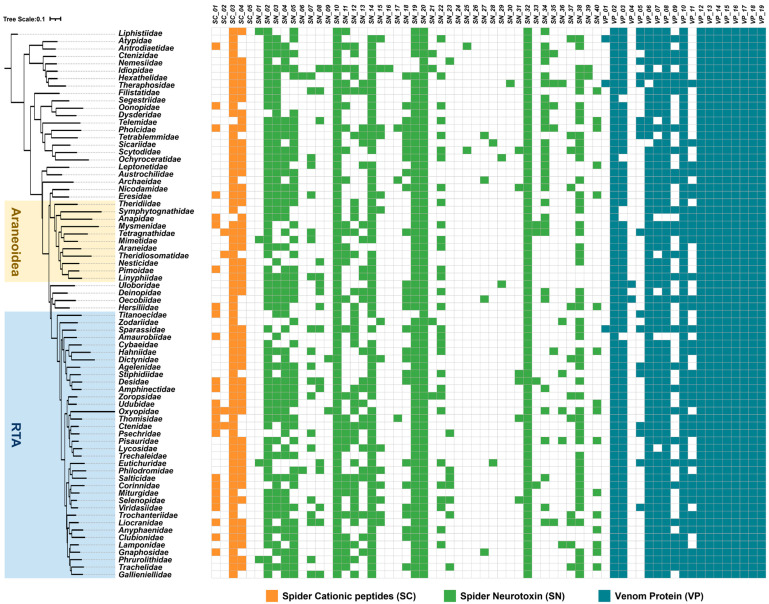
The distribution of venom protein families in Araneae. **Left**: The evolutionary tree of Araneae, with Araneoidea and RTA clades marked in yellow and blue, respectively. **Right**: Statistical overview of venom protein families. The presence of color indicates the presence of family, while the absence of color indicates the absence of family. Brown: spider cationic peptides (SCs); green: spider neurotoxins (SNs); cyan: venom proteins (VPs).

Our results show that spiders share several protein families, including enzymes and large proteins, such as peptidase M12A, phospholipase D, phospholipase A2, angiotensin-converting enzyme, peptidylglycine alpha-amidating monooxygenase, signal peptidase, venom serine protease, venom Kunitz-type family, cysteine-rich secretory protein (CAP superfamily), thyroglobulin-like protein, leucine rich peptide, protein disulfide-isomerase, tachylectin 5A, cystatin, and latrotoxin superfamily (Figure 1 and Table S2). Several types of neurotoxins are also widely distributed throughout Araneae, including huwentoxin-1, CsTx superfamily, plectoxin superfamily, Magi-1 superfamily, and MIT-like AcTx family. These proteins have been associated with a range of activities, including protein synthesis and modification, dissolving cell membrane, acting as inhibitors of crucial ion channels and enzymes, functioning in concert with other toxins, and so on.

Most neurotoxins are distributed specifically in different spider taxa, for example, the potassium channel inhibitor phrixotoxin family, which is specifically present in Theraphosidae [45]. The insecticidal toxin neurotoxin 16 (SFI) family is peculiar to ancient spider species, which can inhibit voltage sodium channels (Nav) in insects [46,47]. The Double-knot toxin family, which is abundant in RTA clade spiders, can activate the capsaicin- and heat-sensitive channel, TRPV1, by targeting the outer pore domain [48,49,50]. In addition, some neurotoxins undergo repeated loss and gain, which may be important for them to adapt to their ecology.

These findings highlight the similarities and differences in the distribution of spider venom protein superfamilies. Throughout the evolutionary process of spider venom, essential enzymes and large proteins remain conserved from ancestral species, while neurotoxins evolve with specificity. Neurotoxins found within spiders are predominantly rich in disulfide-rich peptides (DRPs). These peptides evolve from a common molecular template [3], possibly resulting in the acquisition of new activities through the modification of other functional residues outside the core template.

### 2.2. High Expression of the CAP Superfamily in Araneoidea

We collected publicly available high-quality genomes from 14 spiders and 1 scorpion (Arachnida), as well as venom gland transcriptomes from 6 spiders and 1 scorpion (Table 1).

**Figure 2:**
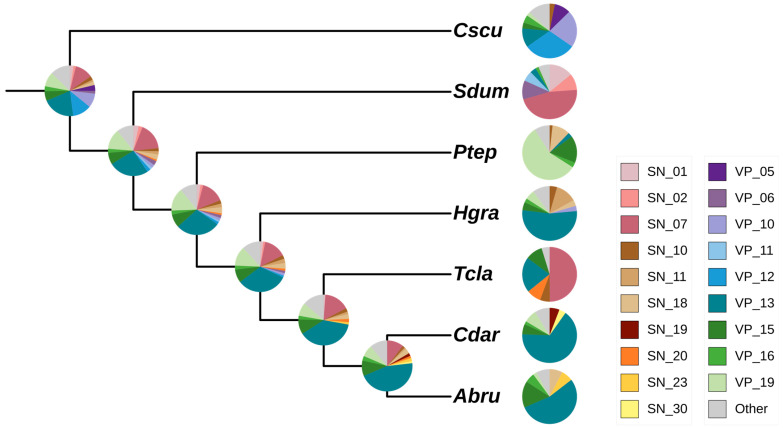
Ancestral state reconstruction of the venom expression phenotype. The species abbreviations are detailed in Table 1. The pie chart shows the expression proportion of each venom protein family. The different venom protein families are represented by different colors.

We analyzed the venom expression phenotype of 6 spiders using the scorpion as an outgroup. The venom proteins are classified into 18 main venom protein families and an “Other family” group. In all examined spiders, the venom composition is characterized by a primary representative family, along with several secondary families. The single predominant family constitutes approximately 50% or more of the total venom expressed (Figure 2). For example, in *Parasteatoda tepidariorum*, the core venom family belongs to the latrotoxin superfamily (VP_19), which is known for its ability to stimulate neurotransmitter release [64]. This specific high expression is attributed to the lineage-specific duplication of latrotoxin genes within Theridiidae [54]. *Trichonephila clavate* and *Stegodyphus dumicola* exhibit elevated expression levels of the beta/delta agatoxin family (SN_07), which is an insecticidal neurotoxin family that modulates the insect Nav channel [65]. Our findings provide the first description of the presence of this neurotoxin in these two spiders.

The CAP superfamily (VP_13) demonstrates a significant expression within Araneoidea (Linyphiidae and Araneidae) and exhibits widespread presence within the Araneae taxa (Figure 1). We regarded the expression proportion of the specific venom protein family as a continuous phenotype and reconstructed the ancestral state by Phytools package. According to ancestral state reconstruction, the high expression of the CAP superfamily can be traced to a common ancestor of spiders and the scorpion, followed by distinct expression patterns during differentiation into diverse spiders. Notably, certain spiders such as *Hylyphantes graminicola*, *Caerostris darwini*, and *Argiope bruennichi* show high expression levels, suggesting variations in their evolutionary trajectories.

Although we identify a large number of toxin genes in the spider genomes, most spiders prefer to use a single venom protein family as their primary toxin, which accounts for more than 50% of the expression. At the same time, there are several other toxins with relatively low expression. These results demonstrate the influence of gene expression on trait complexity and diversity.

### 2.3. Identification and Subfamily Classification of the CAP Superfamily

**Figure 3:**
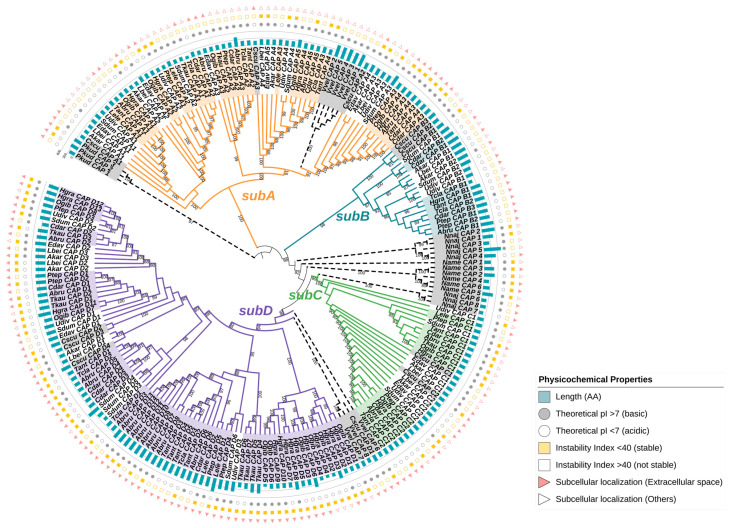
Phylogenetic analysis of the CAP superfamily. Evolutionary tree of CAP superfamily members of 14 spiders, 1 scorpion (Cscu), 1 fungus (Pkud), 1 roundworm (Name), 1 insect (Vvel), and 1 snake (Nnaj). The branches of different subfamilies are represented by different colors. Orange: subA; cyan: subB; green: subC; purple: subD. The branches of the fungi, roundworms, insects, and snakes are marked with dotted lines. The protein ID ranges of Araneoidea are labeled with subfamily colors, and those of non-spider species are labeled with gray. The outer circle from inside to outside shows the length of the amino acid sequence (AA), theoretical pI, instability index, and subcellular localization of each protein.

We conducted further identification, structural, and evolutionary analyses of the CAP superfamily. A total of 189 members were identified in 14 spiders and 1 scorpion, ranging from 6 to 22 per species (Figure 3 and Table S5). *Argiope bruennichi* and *Hylyphantes graminicola* have the most abundant CAP members, while *Latrodectus elegans* has the least. The number of CAP superfamily members in each species is consistent with gene expression levels. Since the evolutionary relationship between the CAP proteins of spiders and scorpions is very close, we conducted a comprehensive analysis of the relationships between CAP proteins in one fungus (*Pichia kudriavzevii*, Pkud), one roundworm (*Necator americanus*, Name), one insect (*Vespa velutina*, Vvel), one snake (*Naja naja*, Nnaj), and spiders (Table S4). Since the C-terminal domain of the CAP protein is highly variable, the conserved CAP domain shared by each protein was used to construct the maximum likelihood (ML) tree (Figure 3 and Figure S1). We selected the fungal CAP proteins as an outgroup to adjust the root node. The phylogenetic analysis revealed four CAP subfamilies of spiders and one scorpion that differ in structure, namely, subA to subD, with subC and subD being sister clades. Among them, subD has the most abundant membership with a total of 84 members, followed by subA with 58 members, subC with 27 members, and subB with 20 members (Table S6).

CAP proteins are made up of approximately 117 to 575 amino acids, with molecular weights ranging from 13 to 64 kDa (Table S6). Notably, the grand average of hydropathy (GRAVY) for these CAP proteins consistently exhibits negative values, indicating their hydrophilic nature. The instability index reveals that subD predominantly exhibits values below 40, suggesting its inherent stability. Conversely, subB elevates the instability index exceeding 40, implying a potential propensity for instability. With website tools BUSCA, we predicted the subcellular localization of these proteins. Of the 189 CAP proteins, 123 are predicted to be localized in the extracellular space, while other members are predicted to be in the cytoplasm, nucleus, and plasma membrane, with subA predominantly localized in the cytoplasm.

The CAP proteins of spiders and scorpions exhibit a close evolutionary relationship, indicating that the divergence within the CAP subfamilies may have occurred in the common ancestors of spiders and scorpions. Compared to other venomous animals, spiders have the most abundant CAP subfamilies. The phylogenetic analysis revealed that subA is closely related to CAP proteins found in ancient fungi. On the other hand, subB seems to be an independent clade and only found in a few spider species, indicating a potential loss of this subfamily in certain lineages. Notably, subC forms a sister branch to snake CAP proteins, while subD is closely related to insect CAP proteins. This suggests that the various CAP superfamily members of spiders seem to be in a transitional phase.

### 2.4. The Domain and Motif Composition of the CAP Superfamily

**Figure 4:**
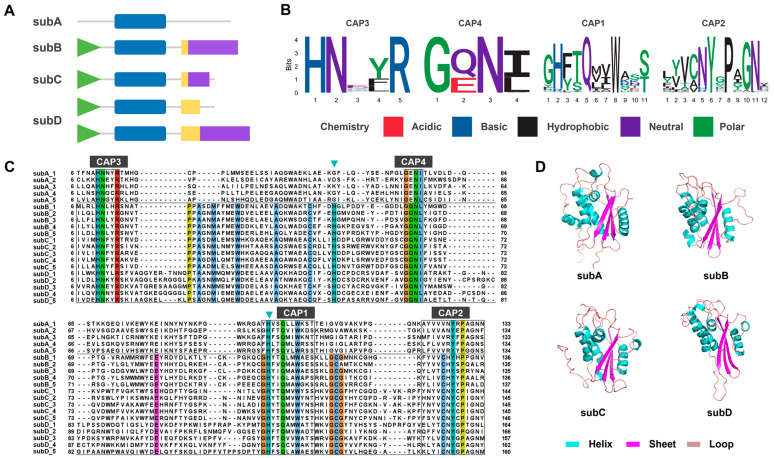
Comparison of CAP domains among four CAP subfamilies. (**A**) Structural diagram of each subfamily. Signal peptide: green triangle; CAP domain: blue rectangle; Hinge domain: yellow rectangle; ICR domain: purple rectangle. (**B**) Seq logos of four conserved CAP motifs. The size of each letter corresponds positively to the frequency of occurrence of that specific amino acid type at the given location. (**C**) Multiple sequence alignment of CAP domains, five proteins of each subfamily are selected. Conserved sites are marked with a Clustal color style. Conserved His sites that bind to bivalent cations are marked with blue triangles. (**D**) Three-dimensional structure of CAP domains of each spider CAP subfamily.

Based on the domain analysis, it can be observed that all members of the CAP superfamily possess the CAP domain (Figure 4A), and its sequence and spatial structure are conserved. Multiple sequence alignment results revealed four conserved motifs of the CAP domain, namely, CAP3, CAP4, CAP2, and CAP1 (Figure 4B,C). The CAP domain exhibits an α-β-α fold spatial structure containing three β-sheets and five to six α-helixes (Figure 4D). Tadokoro et al. demonstrated that the central lumen of the CAP domain contains two conserved histidine residues that can serve as metal ion binding sites [17]. We notice that the two histidine residues exist in most CAP proteins, but one is missing in subA (Figure 4C). Given the apparent evolutionary status of subA, we further suggest that it may have functional differences from the other three subfamilies. In addition, except for subA, the other three CAP subfamilies contain C-terminal domains (Figure 4A). Specifically, subB, subC, and part of subD consist of the signal peptide, the conserved CAP domain, hinge domain, and ICR domain, while the other part of subD lacks the ICR domain.

Our phylogenetic analysis revealed high similarity between spider CAP subD and insect CAP superfamily members, as well as between subC and snake members (Figure S1). To investigate the potential function of spider CAP proteins, we compared them with known functional CAP proteins in snake and insect venoms. Utilizing the Tox-Prot database, we retrieved insect venom allergen 5 (Ag5) proteins which cause allergic reactions, and snake svCRISP protein which acts on ion channels.

**Figure 5:**
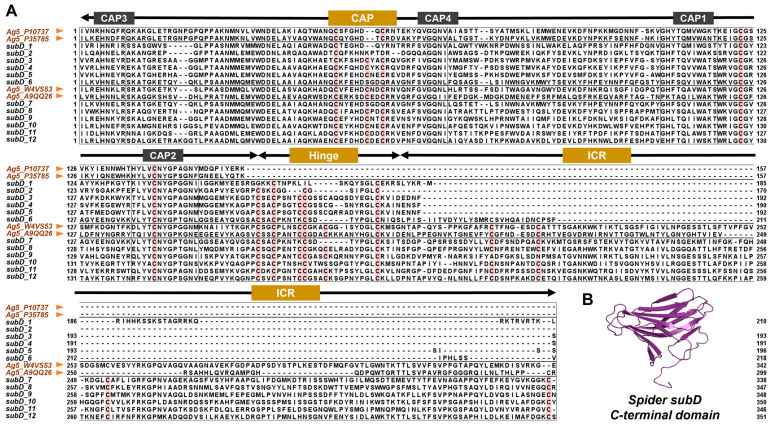
Comparison of spider CAP subD and insect venom allergen 5 (Ag5). (**A**) Multiple sequence alignment between spider CAP subD and insect Ag5. Ag5 proteins are indicated by orange arrows, and the conserved cysteine residues are marked in red. (**B**) Three-dimensional structure of the unique C-terminal domain of spider CAP subD members.

On the one hand, we selected 4 insect Ag5 proteins that possess different C-terminal domains, along with 12 spider CAP subD proteins for alignment analysis. The spider CAP subD proteins can be divided into two groups. The first group (subD1–6) has a cysteine framework similar to insect Ag5, while the second group (subD7–12) possesses a unique C-terminal domain (Figure 5A). The unique C-terminal domain identified in this study is distinct from all known functional domains. It contains four conserved cysteine residues and is capable of forming a poly β-sheet structure (Figure 5B), which indicates that this type of CAP subD can perform a specific biological function in spiders.

**Figure 6:**
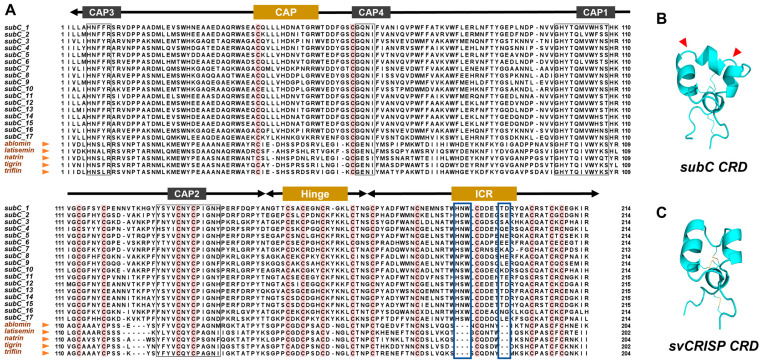
Comparison of spider CAP subC and snake svCRISP. (**A**) Multiple sequence alignment between spider CAP subC and snake svCRISP. The svCRISP proteins are indicated by orange arrows, and the conserved cysteine residues are marked in red. The two extra short fragments are marked with blue boxes. (**B**) Three-dimensional structure of subC CRD domain, with the red arrows pointing to the spatial position of the two extra short fragments. (**C**) Three-dimensional structure of snake svCRISP CRD domain.

On the other hand, we selected 5 svCRISP proteins (*ablomin*, *latisemin*, *natrin*, *tigrin*, and *triflin*) from snakes that possess the C-terminal ShKT domain, and 17 CAP subC proteins from spiders for alignment analysis. The svCRISP proteins found in snake venom are an essential part of the CAP superfamily, whose functions are well researched. These proteins feature an ShKT domain on the C-terminal, which enables them to regulate various ion channels [34,35,40]. Spider CAP subC proteins have a similar C-terminal structure consisting of a hinge domain with four conserved cysteine residues and an ICR domain with six cysteine residues. However, the function of these CAP proteins in spiders is unknown. The alignment results show that the sequence similarity between spider CAP subC and svCRISP is very high, but spider subC has two more short fragments in its ICR domain (Figure 6A), which separate three α-helix into four α-helix in spatial (Figure 6B,C). By comparing these two types of proteins, we suggest that spider CAP subC has a potential function as an ion channel regulator, but changes in the three-dimensional structure may lead to functional differences.

### 2.5. Gene Duplication and Evolution of the CAP Superfamily

**Figure 7:**
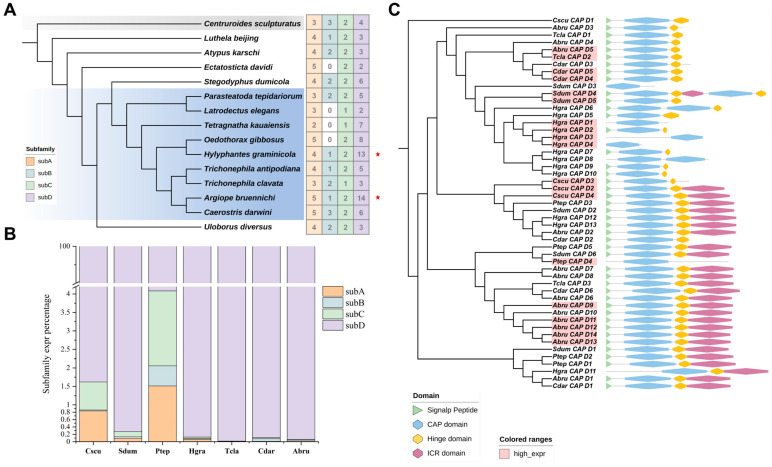
The copy number, expression, and domain analysis of CAP subfamilies. (**A**) The copy number of CAP subfamily members per species. The red stars mark spiders with more than 10 copies of CAP subD. The gray and blue background colors represent the outgroup and Araneoidea respectively. (**B**) The expression ratio of four subfamilies in 7 species. (**C**) Phylogenetic relationship and domains of CAP subD proteins. The labels highlighted in red are highly expressed CAP genes (tpm > 100).

The CAP superfamily is highly expressed in several species of Araneae (Figure 2). Given the rich diversity of members in the spider CAP superfamily, we aim to further investigate which members play a key role. Through the analysis of gene copy numbers and expression levels across four subfamilies, our focus narrows down to subD, which has more than ten copies in two spiders and contributes to over 90% expression of all genes (Figure 7A,B). However, genes belonging to other subfamilies have minimal expression values. It is worth noting that partial CAP genes of subD are exceptionally highly expressed (tpm > 100) (Figure 7C, Table S7). In *Hylyphantes graminicola* and *Trichonephila clavata*, one or more subD genes are specifically upregulated in the venom gland (Figure S2).

Amplified toxin genes form a gene family that can increase the expression of toxins to aid in the replenishment of venom [66]. Particularly, *Argiope bruennichi* has seven highly expressed CAP subD genes, which can explain the highest proportion of CAP superfamily at its transcriptomic (Figure 2) and proteome levels [26]. These highly expressed subD members are observed to possess different C-terminal domains (Figure 7C). The first has a short C-terminal domain similar to the insect Ag5 that may have the ability to cause allergic reactions. The second has a long C-terminal domain with eight conserved cysteine residues, which is unique in spiders (Figure 5B). These results indicate that the spider CAP subD genes have the potential to function as venom proteins regardless of the integrity of the C-terminal domain.

Compared to Mollusca [67,68] and Reptilia [16,20], the core CAP superfamily in Arthropoda forms a distinct group. Although spiders possess subC, which is similar to more toxic svCRISP, they specifically express subD, which is homologous to Ag5 found in Hymenoptera insects. Natural selection made the CAP superfamily the core toxin of the web-building spiders whose venom can only paralyze prey but lacks lethal effects [26]. This suggests that the CAP subD selected by spiders may play a crucial role in paralyzing prey.

**Figure 8:**
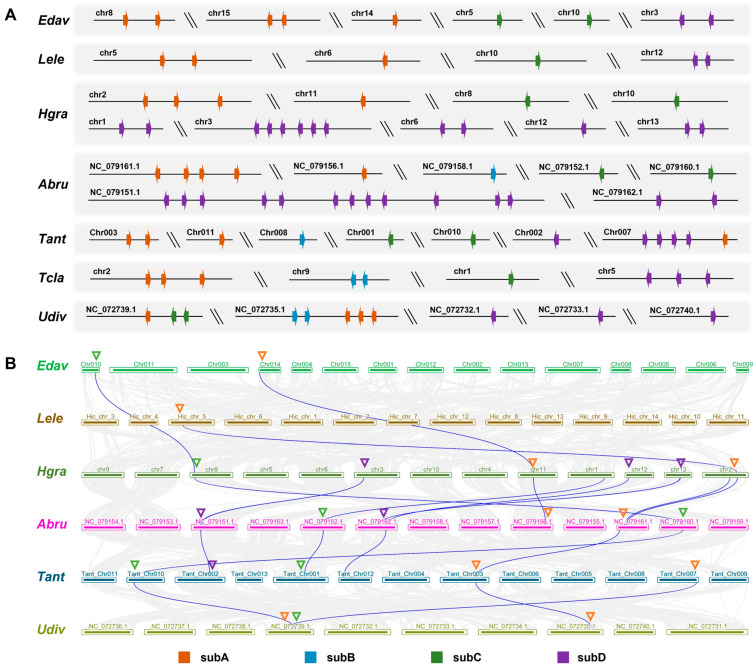
Chromosome positions and collinearity of CAP genes. (**A**) CAP gene array of each species on the chromosome; different subfamilies are represented by arrows of different colors (orange: subA; blue: subB; green: subC; purple: subD). (**B**) The collinear analysis of 6 spiders. CAP genes are highlighted with thin blue lines. The CAP subfamily sites are marked with triangles in the same color as (**A**).

Finally, we analyzed the evolutionary process of the CAP superfamily in Araneae by examining the chromosomal locations of the genes and collinearity analysis across species. It can be observed that the expanded subD genes are mostly clustered on the same chromosome (Figure 8A). Additionally, there is always a collinearity in CAP genes between different species (Figure 8B). These collinear gene pairs are highly similar to sequences and belong to the same subfamily. The subA collinearity exists in all species, and it is the oldest CAP subfamily in spiders (Figure S1), indicating that the position of the CAP gene on the genome is relatively conserved and can be stably preserved during evolution. With the exception of *Latrodectus elegans* (Theridiidae), subC also displays collinearity in different spiders. Although CAP subA and subC are stably inherited, their expression levels are extremely low.

The collinearity of subD is only shown in Araneoidea, which has the most copies of subD. Among the four subfamilies, spiders prefer to select subD as the efficient members. They produce multiple copies of subD through duplication events, particularly in Araneoidea, and the coexpression of these clustered genes greatly increases the expression level of the CAP superfamily in venom. These results indicate that different CAP subfamilies existed since the spider ancestor. During evolution, some subfamilies undergo loss, retention, or duplication, with gene duplication potentially serving as the primary driving force. Amplified CAP genes are then retained in closely related species.

## 3. Discussion

We calculated the distribution of spider venom families using transcriptome data from 74 spiders. We found that most spiders share many enzymes and large venom proteins, while neurotoxins show a high degree of lineage specificity. The enzymes present in venom can cause cell damage, trigger the release of immune molecules, lead to inflammation, and, subsequently, induce pain and swelling in the prey [69]. On the other hand, neurotoxins act on various ion channels, disrupt neuronal function, and rapidly paralyze the prey [70]. Conserved enzymes and various neurotoxins work together to maintain the basic function of the venom, while also helping the spiders adapt to different ecology.

Variations in gene expression contribute to the complexity and diversity of traits. The expression of venom protein families suggests that spiders have their specific types or combinations of toxins for predation or defense. For better adaptation to the ecology and hunting, spider venom has undergone independent evolutionary trajectories. Typically, the venom of most spiders comprises a single, pivotal toxin, accompanied by several auxiliary toxins. This composition not only guarantees the fundamental function of the venom but also affords spiders the potential to adapt to diverse ecology. Barua et al. demonstrated that there is no limit to the evolution of toxin combinations, but most snakes prioritize either a single or a combination of major toxin families [71]. Here, we observe that the CAP superfamily is a core toxin found in various species of Araneoidea. It is a venom protein family that was initially identified as a venom component in snakes and Hymenoptera. The structure, evolution, and function of the CAP superfamily in spiders remain understudied. Our study provides further analysis of this toxin which is overlooked due to its abundance in these web-building spiders.

We identified four different subfamilies of the CAP superfamily, which were named subA–subD. Each subfamily has its specific domain composition. The conserved CAP domain is shared by all subfamilies. The central cavity of the CAP domain can bind to metal ions, which may regulate the function of the protein [17]. However, the function of the C-terminal domain is unknown. By comparing the CAP protein sequences with those of snakes and insects, we interpret the potential ion channel regulation of spider CAP subC and the ability of CAP subD to induce allergic reactions. In addition, we also report a new group of CAP superfamily with a unique C-terminal domain composed of β-sheet. The potential function of this domain deserves further exploration.

Gene duplication and adaptive selection are thought to be the main drivers of venom evolution [72,73]. Venom genes expand to form the gene family that can coexpress and lead to a significant increase in expression level. Following the evidence of the latrotoxin superfamily in Theridiidae [72], our findings demonstrate gene duplication of the CAP superfamily. The high expression of one or more CAP subD genes makes the CAP superfamily core component in the venom of certain Araneoidea spiders, including *Argiope bruennichi*, *Caerostris darwini*, and *Hylyphantes graminicola*. This core toxin plays a crucial role in enabling these web-building spiders to effectively paralyze their prey.

## 4. Conclusions

Our findings reveal the distribution characteristics of spider venom protein families. Most spiders have conserved enzymes and lineage-specific neurotoxins that work together to maintain basic venom function and help spiders adapt to the ecology. The differences in the expression of venom protein families reflect the adaptive strategies of spider predation or defense. By demonstrating the structure, evolution, and function of the CAP superfamily, our results help to understand the specific, highly expressed toxins in the overlooked web-building spiders’ venom. The CAP superfamily is speculated to be related to ion channel regulation and allergic reactions, which help spiders paralyze their prey. Our study also shows that gene duplication and adaptive selection are the main drivers of the evolution of the CAP superfamily, with coexpression of genes giving it a core status in the venom. These findings not only deepen our understanding of the complexity and diversity of spider venom, but also provide a basis for further research into the function and evolutionary mechanisms of spider venom.

## 5. Materials and Methods

### 5.1. De Novo Transcriptome Assembly and Annotation

We retrieved spider transcriptome data published by Arakawa et al. [44]. The raw reads were quality-controlled using fastp v0.23.1 [74], and the cleaned reads were assembled by Trinity v2.14.0 [75]. Redundant sequences were removed using cd-hit-est v4.8.1 [76] (-c 0.95, -n 10). To minimize the presence of false positives in the transcript assembly, each transcript was quantified by salmon v1.9.0 [77], and sequences with transcripts per million (TPM) ≤ 1 were filtered out. We conducted the prediction of the long open reading frame (ORF) for each transcript using TransDecoder v5.7.0 [78]. Then, we identified ORFs with homology by searching against the UniProtKB/Swiss-Prot and Pfam databases based on BLASTP v2.12.0 [79] (E-value ≤ 1 × 10^−3^) and HMMER v3.3.2 [80]. After selecting the longest transcripts, we validated the annotation completeness using BUSCO v5.4.6 [81] (arthropoda_odb10).

### 5.2. Toxin Gene Identification and Classification

We initially collected 7718 manually annotated animal venom secreted proteins from Tox-Prot [82] and 1838 spider toxin proteins from ArachnoServer 3.0 [4]. We filtered out candidate toxin genes using BLASTP v2.12.0 [79] (E-value ≤ 1 × 10^−5^) by conducting homology alignment on these two datasets. To classify these candidates, we used HMMcompete v0.1 [43], which incorporates 64 hidden Markov models (HMMs) consisting of well-known spider venom protein superfamilies. Finally, we counted the members of each superfamily and visualized the data with iTOL v6.9 [83] (https://itol.embl.de/, accessed on 20 October 2023).

### 5.3. Ancestral State Reconstruction of the Spider Venom Phenotype

For phylogenetic analysis, the orthologous genes were identified using Orthofinder v2.5.4 [84] (-S blast -M msa). All single-copy orthologous genes were then aligned by Muscle5 [85] and trimmed by trimAl v1.4 [86] (-automated 1). The trimmed sequences were concatenated to form a “Supergene” for constructing the species tree using IQtree2 [87] (-m MFP -B 1000) with the scorpion as an outgroup. The species divergence time was obtained from Timetree (https://timetree.org/, accessed on 20 October 2023), and the ultrametric tree was constructed using r8s v1.8.1 [88]. The proportion of specific venom protein families expressed was considered a continuous phenotype [89], and ancestral state reconstruction was performed using the Phytools v2.0-3 package [90] with the BM model.

### 5.4. CAP Superfamily Identification and Evolution

To identify potential members of the CAP superfamily, we conducted a thorough filtering process. We started by using HMMER v3.3.2 [80] to search for domains within each candidate. Subsequently, candidates lacking the CAP (PF00188) or crisp (PF08562) domain were filtered out. Next, we applied FIMO in MEME v5.5.3 [91,92] to search the four known motifs (CAP1-CAP4), and manually screened to confirm members containing 3 or more motifs. Finally, signalp6 [93] (-org euk) was utilized to identify the signal peptide of each protein. The subcellular localization and protein physicochemical properties of CAP superfamily members were analyzed by the website tools BUSCA [94] (https://busca.biocomp.unibo.it/, accessed on 20 October 2023) and ExPASy ProtParam [95] (https://web.expasy.org/protparam/, accessed on 20 October 2023).

To construct a reliable evolutionary tree for the CAP superfamily, we extracted the conserved CAP domain from each protein and used it as the input sequence. We conducted phylogenetic analysis using Muscle v5.1 [85] and IQtree v2.2.5 [87], and classified four subfamilies based on the resulting tree. Afterwards, we renamed each identified protein accordingly. Jalview v2.11.3.0 [96] was utilized to visualize the results of multiple sequence alignment. Furthermore, we predicted the 3D structure for a representative member of each subfamily by Alphafold2 [97] and imported the PDB file into PyMOL v2.5.7 for visualization.

### 5.5. Transcriptome Analysis

We retrieved publicly available transcriptome data from the venom glands of six spiders and one scorpion, as well as data obtained from various tissues of two spiders (*Hylyphantes graminicola* and *Trichonephila clavata*), which can be found with their SRA accession numbers in Table S3. To ensure quality, the raw sequencing reads were initially subjected to fastp v0.23.1 [74]. The resulting clean reads were then aligned to the reference genome using STAR v2.7.9a [98]. Quantification was performed with featureCounts v2.0.3 [99] to count the number of reads mapped to each gene.

### 5.6. Chromosomal Localization and Genome Collinearity Analysis

The positional information of CAP genes is derived from the GFF file, followed by the visualization of each gene on its corresponding chromosome. We performed a genomic collinearity analysis of six spiders with chromosome-level genomes distributed in different taxa. The MCScanX program of TBtools v2.095 [100] was utilized for the identification and visualization of genomic collinearity.

## Figures and Tables

**Table 1:** Genomes of spiders and a scorpion used in this paper.

Family	Species	Abbr.	Link/Accession	Assembly	Reference
Buthidae	*Centruroides sculpturatus*	Cscu	GCF_000671375.1	scaffold	PRJNA168116
Liphistiidae	*Luthela beijing*	Lbei	https://doi.org/10.57760/sciencedb.07403 (accessed on 18 October 2023)	scaffold	(Jin et al., 2023) [51]
Atypidae	*Atypus karschi*	Akar	https://doi.org/10.57760/sciencedb.07403 (accessed on 18 October 2023)	scaffold	(Jin et al., 2023) [51]
Hypochilidae	*Ectatosticta davidi*	Edav	https://doi.org/10.57760/sciencedb.06872 (accessed on 20 February 2023)	chromosome	(Fan et al., 2023) [52]
Eresidae	*Stegodyphus dumicola*	Sdum	GCF_010614865.2	scaffold	(Liu et al., 2019) [53]
Theridiidae	*Parasteatoda tepidariorum*	Ptep	GCF_000365465.3	scaffold	(Gendreau et al., 2017) [54]
Theridiidae	*Latrodectus elegans*	Lele	http://dx.doi.org/10.5524/102210 (accessed on 20 February 2023)	chromosome	(Wang et al., 2022) [55]
Linyphiidae	*Oedothorax gibbosus*	Ogib	GCA_019343175.1	chromosome	(Hendrickx et al., 2022) [56]
Linyphiidae	*Hylyphantes graminicola*	Hgra	https://doi.org/10.11922/sciencedb.01162 (accessed on 20 February 2023)	chromosome	(Zhu et al., 2022) [57]
Tetragnathidae	*Tetragnatha kauaiensis*	Tkau	https://doi.org/10.5061/dryad.b2rbnzsgr (accessed on 20 February 2023)	scaffold	(Cerca et al., 2021) [58]
Araneidae	*Trichonephila antipodiana*	Tant	http://dx.doi.org/10.5524/100868 (accessed on 20 February 2023)	chromosome	(Fan et al., 2021) [59]
Araneidae	*Trichonephila clavata*	Tcla	https://spider.bioinfotoolkits.net/ (accessed on 20 February 2023)	chromosome	(Hu et al., 2023) [60]
Araneidae	*Argiope bruennichi*	Abru	GCF_947563725.1	chromosome	(Sheffer et al., 2021) [61]
Araneidae	*Caerostris darwini*	Cdar	GCA_021605075.1	scaffold	(Babb et al., 2022) [62]
Uloboridae	*Uloborus diversus*	Udiv	GCF_026930045.1	chromosome	(Miller et al., 2023) [63]

## Data Availability

Data are contained within the article and Supplementary Materials.

## Supplementary Materials

The following supporting information can be downloaded at: https://www.mdpi.com/article/10.3390/toxins16060240/s1, Figure S1: Phylogenetic relationship of the CAP superfamily between spiders and outgroups; Figure S2: Multi-tissue expression analysis of CAP genes in two spiders. (A) *Hylyphantes graminicola*. (B) *Trichonephila clavate*; Table S1: Publicly available global transcriptome data used in this paper; Table S2. Identification results of 64 spider venom protein families; Table S3: Publicly available venom gland transcriptome data used in this paper; Table S4: Publicly available genome data from four outgroups; Table S5: Identification and screening of the CAP superfamily from 14 spiders and 1 scorpion; Table S6: Physicochemical properties and subcellular localization of CAP superfamily members; Table S7: TPM of CAP superfamily members.
