# Supplementary figures and images for: Identification and Evolutionary Analysis of the Widely Distributed CAP Superfamily in Spider Venom
Source: Toxins (Basel). 2024 May 24;16(6):240. doi: 10.3390/toxins16060240 (PMC11209345; doi:10.3390/toxins16060240)

Tree scale: 0.1

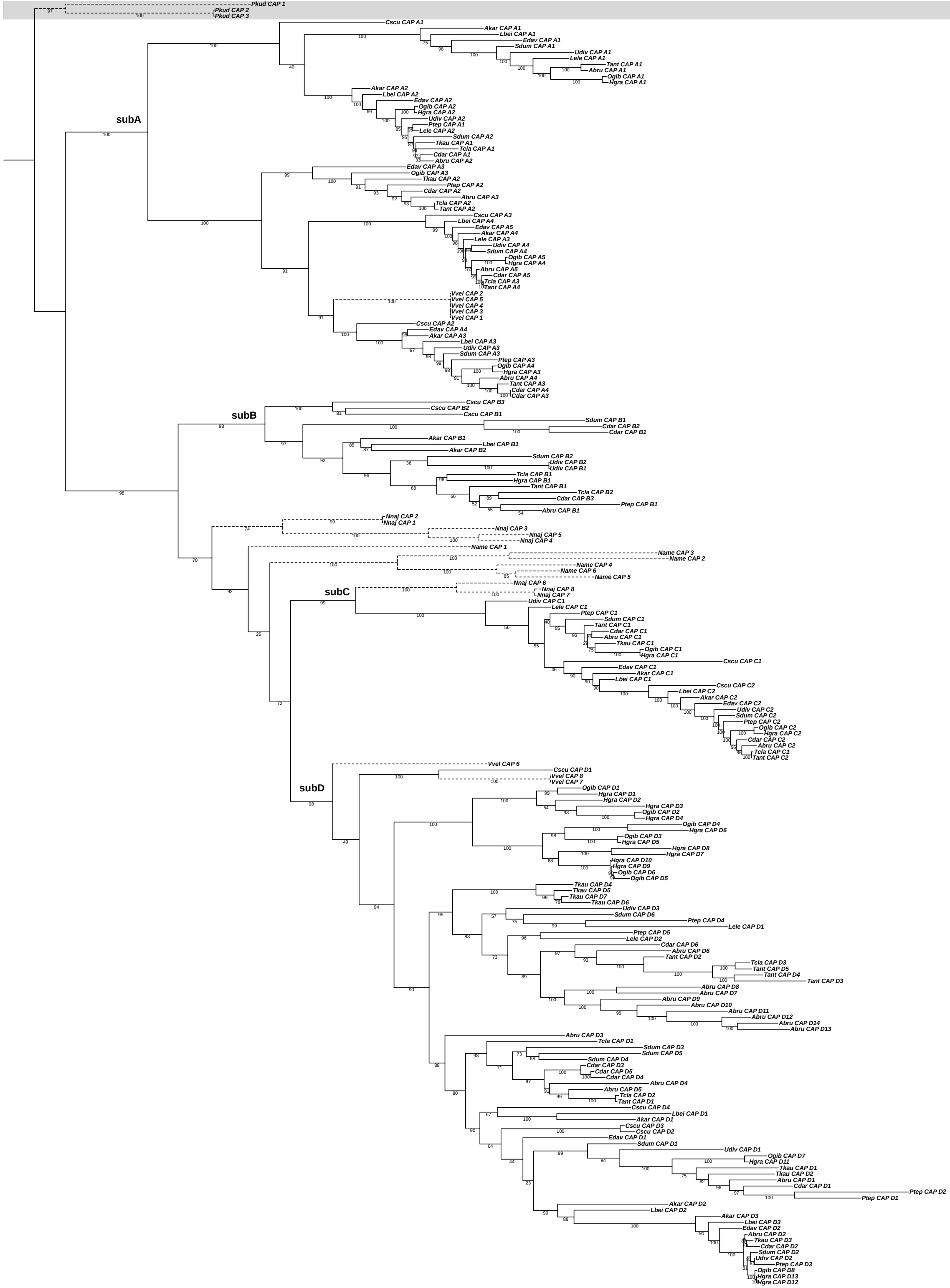

Supplement: Supplementary file 1 [file toxins-16-00240-s001.zip › FigureS1.pdf]

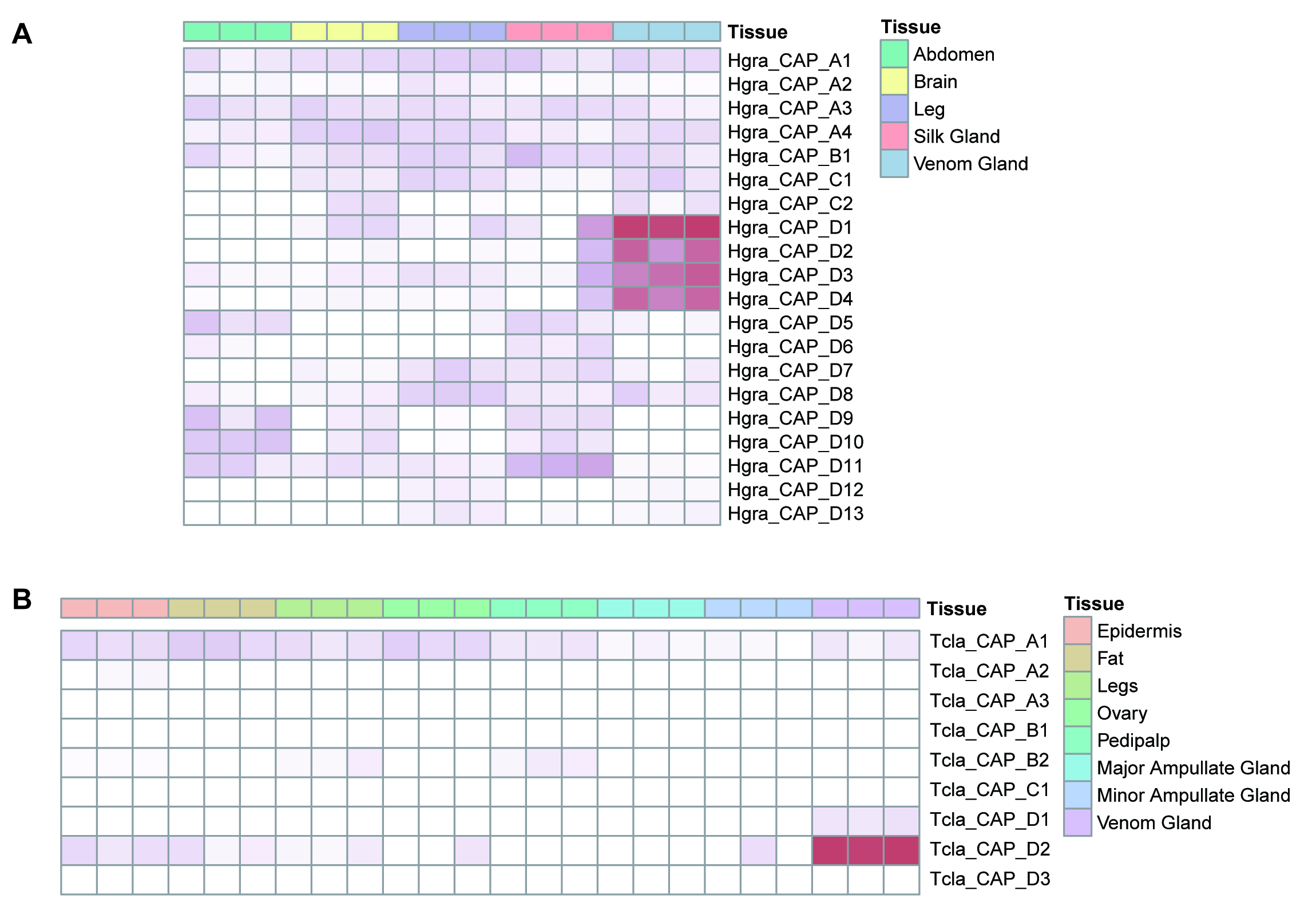

Supplement: Supplementary file 1 [file toxins-16-00240-s001.zip › FigureS2.tif]
